# Hematological and Biochemical Reference Intervals for 5 Adult Hunting Dog Breeds Using a Blood Donor Database

**DOI:** 10.3390/ani10071212

**Published:** 2020-07-16

**Authors:** Arianna Miglio, Alessandra Gavazza, Donatella Siepi, Francesco Bagaglia, Ambra Misia, Maria Teresa Antognoni

**Affiliations:** 1 Department of Veterinary Medicine, Blood Bank and Transfusion Unit EMOVET-UNIPG, University of Perugia, 06126 Perugia, Italy; ambralisa@hotmail.it (A.M.); maria.antognoni@unipg.it (M.T.A.); 2 School of Bioscences and Veterinary Medicine, University of Camerino, 62024 Camerino, Italy; alessandra.gavazza@unicam.it; 3 Department of Medicine, University of Perugia, 06124 Perugia, Italy; donatella.siepi@unipg.it (D.S.); francesco.bagaglia@alifax.com (F.B.)

**Keywords:** blood donors, breed, hunting dogs, hematology, biochemistry, reference intervals

## Abstract

**Simple Summary:**

Hematological and biochemical profiles are essential in the diagnosis and monitoring of disease in veterinary medicine, requiring optimal Reference Intervals (RIs) for accurate interpretation. The aim of this study is to determine hematobiochemical RIs for 5 hunting dog breeds from a blood donor database and to compare them with laboratory established and published RIs to identify possible breed and attitude-related differences. A total of 445 healthy adult hunting dogs (156 Ariégeois, A; 52 Bleu de Gascogne, B; 64 Bracco italiano, C; 123 Segugio italiano, D; 50 Briquet Griffon Vandeen, E) were included in the study. Significant differences in 12 hematologic and serum biochemical analytes, for which a breed-specific variation appears to be the most plausible explanation, were detected, and new RIs for these parameters are provided.

**Abstract:**

Numerous studies have shown the importance of breed-related differences between hematological and biochemical results in veterinary medicine. The aim of this study is to determine hematologic and biochemical Reference Intervals (RIs) for 5 hunting dog breeds from a blood donor database, adopting an indirect sampling method, and to compare them with laboratory established and published RIs to identify possible breed and attitude-related differences. The study analyzed the blood parameters of 445 adults (222 females and 223 male, with age ranging from 2 to 8 years, mean age 5.3 years), client-owned, clinically healthy blood donor dogs of 5 breeds: 156 Ariégeois, 52 Bleu de Gascogne, 64 Bracco italiano, 123 Segugio italiano, and 50 Briquet Griffon Vandeen. Statistical analysis was performed as recommended by the American Society of Veterinary Clinical Pathology (ASVCP) guidelines. RIs for red blood cells (RBC), hematocrit (HCT), hemoglobin (HB), main corpuscular volume (MCV), main corpuscular hemoglobin (MCH), main corpuscular hemoglobin concentration (MCHC), red distribution widht (RDW), white blood cells (WBC), and differential leukocytes count, PLT, Albumin, Total Protein, Urea, Creatinine, aspartate aminotransferase (AST), alanine aminotransferase (ALT), and alkaline phosphatase (ALP) for each of the 5 breeds were performed, and significant differences with the established RIs were detected. We found significant differences in 12 hematologic and serum biochemical analytes for which a breed-specific variation appears to be the most plausible explanation. New RIs for HCT, MCH, MCHC, RDW, PLT, Monocytes, Eosinophils, Albumin, Urea, Creatinine, AST, and ALT are provided for at least 1 breed. Breed-specific RIs for adult hunting dogs will help avoid misinterpretation of laboratory results in these breeds.

## 1. Introduction

Hematological and biochemical profiles are essential in the diagnosis and monitoring of disease in veterinary medicine, requiring optimal Reference Intervals (RIs) for accurate interpretation [1,2,3,4,5,6,7,8].

The dog is the most polymorphic terrestrial mammal on the planet, and breed-specific phenotypic diversity examining clinicopathological data has been demonstrated [9,10,11]. Genetic and phenotypic variability, both between canine breeds and populations in different geographic locations of the same breed, are likely to impact the relevance of general canine RIs [9,12]. 

In the light of this data, there is a recent increasing interest in breed-specific canine RIs. Numerous studies have shown the importance of breed-related differences between hematological and biochemical results, even if all measurements fall within the existing RIs [1,2,8,9,10,12,13,14,15,16,17,18,19,20]. Approved guidelines for the establishment, validation, and transference of RIs to a specific breed exist in veterinary medicine [21,22] and have been successfully used to characterize the breed-specific variation of hematological and biochemical analytes [14,17]. They represent a useful preliminary step in determining whether to develop separate breed RIs. Moreover, such studies underline that the influence of age, sex, and neutering status on hematological and biochemical parameters generally transcend breed-specific phenotypes [9,10]. 

Based on genetic studies, all canine breeds can be regrouped in four distinct groups: Asian/Ancient, Herding, Hunting, and Mastiff breeds [11,23,24]. Regarding the hunting group, the breeds included vary based on the type of physical exercise (intense running, sprinting, terrains and distances covered) and tasks of each hunting work involved. It is well known that exercise induces a variety of physiological and laboratorial changes depending on the characteristics of exercise, i.e., duration and intensity, and on the fitness and training level of the athlete [25,26,27]. The results of some parameters might be out of range for trained dogs, depending on the characteristics of the training. Hunting dogs must have an appropriate fitness level because they have to cover large areas of terrain, sometimes in hard climatic or terrain conditions. Therefore, from a medical point of view, it is important to establish these modifications in some laboratory parameters, because they should be differentiated from those derived from disease and exhaustion. The knowledge of these changes is essential for an early diagnosis of lack of performance to assess the impact of different feeding or supplementation strategies and to minimize the risk of exercise-linked diseases, such as exertional rhabdomyolysis, exhaustion, dehydration, heart stroke, and electrolyte imbalances within others [28].

Hematobiochemical differences and RIs have been described in Bernese Mountain dogs [17], Dogue de Bordeaux [12,29], Greyhound [13], Lurchers [14], and other Sighthound breeds [16,19], Shetland Sheepdogs [18], and Beagles [15]. In all these breeds, new Rls have been defined for one or more parameter according to the standard. No exhaustive studies have been carried out on specific hematological and biochemical RIs in hunting dog breeds. 

Adult RIs are representative when the population they refer to is composed of healthy subjects [30], and the use of a blood donor database can ensure this prerogative, since hematology and clinical biochemistry present a fundamental support to evaluate the health status of blood donors in a Veterinary Blood Bank [5,31,32]. 

Since literature advances veterinary clinical pathology practice toward breed-specific RIs, the aim of the study was to evaluate some hematological and biochemical RIs for clinically healthy adult dogs of 5 hunting breeds, to compare them with laboratory established and published RIs existing for the general canine population, and to determine whether they can be validated or rejected in these breeds. 

## 2. Materials and Methods 

The reference population consisted of 445 healthy adult hunting dogs (156 Ariégeois, A; 52 Bleu de Gascogne, B; 64 Bracco italiano, C; 123 Segugio italiano, D; 50 Briquet Griffon Vandeen, E) sampled for voluntary blood donations at the Blood Bank (EMOVET-UNIPG) of a University Veterinary Teaching Hospital (University of Perugia, Central Italy) between January 2015 and January 2017. 

Each dog enrolled in the study was included in the Italian purebred dog population ENCI (Ente Nazionale della Cinofilia Italiana), and the breeders freely participated in the voluntary blood donor program. Dogs were enrolled using the criteria of suitability indicated in the Ministry of Health Italian Guidelines [33,34] (IGLs): Age ranging from 2 to 8 years (mean age 5.3 years), weight > 25 kg (mean weight 27.2kg), vaccinated, and that have not traveled outside Italy or received a blood transfusion previously. A total of 222 were females (82 in A, 25 in B, 31 in C, 61 in D, 23 in E) and 223 males (75 in A, 27 in B, 32 in C, 62 in D, 27 in E). At the time of donation, each animal tested as healthy after an evaluation of anamnesis, a physical examination, hematobiochemical tests, urinalysis (including protein to creatinine ratio: PU/CU), and parasitological examination of feces, as indicated in the IGLs. Moreover, each dog tested negative for serology against *Leishmania infantum, Anaplasma phagocytopylum, Babesia spp., Borrelia burgdorferi, Ehrlichia canis,* and *Rickettsia spp.,* as suggested by the IGLs [33,35].

Blood sampling was performed in all dogs in the early morning when at rest. Hunting activity was stopped at least 2 weeks before sampling. There was no evidence of dehydration, electrolyte imbalances, stress, or muscle disorders in the enrolled dogs. Whole blood (10 mL) was collected from the cephalic vein and placed in a tube containing K3-EDTA (Venoject, Terumo, Italy) and in a plain tube. Plain tubes were centrifuged (2000 g for 10 min) to obtain serum. Analyses were performed within 1 hour from blood collection. On K3-EDTA tubes, a CBC including the leukocyte differential count was performed using a laser hematology analyzer (Sysmex XT-2000iV; Sysmex, Kobe, Japan), validated for canine blood [15], and equipped with a multispecies software. This analyzer combines laser-based flow cytometry and impedance technology. Quality control and calibration were performed weekly with e-check Xe (Sysmex). The following analytes were measured: white blood cells (WBC), neutrophils, lymphocytes, monocytes, eosinophils, basophils, red blood cells (RBC), main corpuscular volume (MCV), hemoglobin (Hb), and platelets (PLT). Hematocrit (HCT), main corpuscular hemoglobin concentration (MCHC), and red distribution width standard deviation (RDW-SD) were calculated automatically by the analyzer. The Sysmex analyzer has just been validated for the leukocyte count. [15,36]. Microscopic blood smear examination was performed for each sample by a clinical pathologist. Platelets counts and differential cell counts obtained by automated analysis were included only when they were validated by microscopic examination by the same expert clinical pathologist.

Selected biochemical parameters were also performed on each serum using an automated chemistry analyzer (Hitachi 904, Boehringer Mannheim and Seac Radim reagents, Biolabo sas, Les Hautes, France) and included Albumin (Alb; bromocresol green method), Total protein (TP; biuret method, bovine albumin 6 g/dL serum as standard), Urea (urease method), Creatinine (Jaffè method), aspartate aminotransferase (AST; kinetic IFCC-International Federation of Clinical Chemistry method), alanine aminotransferase (ALT; kinetic IFCC method), and alkaline phosphatase (ALP; kinetic IFCC method). Specimen quality was recorded at the time of analysis for hemolysis or lipemia. 

The standard RIs in use in our laboratory for the CBC were transferred from previously published RIs for the Sysmex hematology analyzer in dogs [15] and validated on the basis of the healthy adult canine population referred in the last years, following the American Society of Veterinary Clinical Pathology (ASVCP) guidelines [21] and the National Committee for Clinical Laboratory Standards (IFCC-NCLSI) [37,38,39]; similarly, the standard RIs used for clinical chemistry were validated from those published in Kaneko [40].

### Statistical Analysis 

For each analyte, the results obtained in each breed were compared with the internal laboratory canine RIs (claimed RIs). The latter had previously been modified and validated from those already published using the same instrument [15]. Statistical analysis was obtained using a specific software (SPSS, version 22.0; Inc. Chicago, IL, USA) after the removal of data that, according to the Tukey rule, behave as outliers interpretable as aberrant observations [1,3,37,38]. Data were ordered using the RAND function of Microsoft Excel. The first 20 items for each analyte were randomly selected and compared with the internal laboratory RIs. The standard laboratory RIs were validated if 10% or less (0–2) of data fell outside the RI; on the other hand, they were rejected if >25% of data (5 or more) fell outside the RI. If 10–25% of data lay outside the laboratory RIs, the next 20 items on the list were selected and compared with the RIs as described above, using the threshold of 10% of data outside the RIs (*n* = 2) to validate or reject the RIs.

A breed-specific RI was generated using an Excel spreadsheet with the Reference Value Advisor (v2.0) set of macroinstructions [22]. The software performs the following computations recommended by the IFCC-NCLSI [37]: Descriptive statistics (mean, medium, SD, minimum and maximum values), tests of normality (Anderson–Darling with histograms and Q–Q plots and Box–Cox transformation), outlier analysis. Both Dixon–Reed and Tukey tests were used, and outliers classified as suspected suspicious were retained, as recommended by the ASVCP guidelines [21], while far outliers were removed from the analysis. RIs were calculated using standard and robust methods on both non-transformed and transformed data. The software indicates the best method to define the RI based on data distribution.

As recommended by the ASVCP guidelines and the IFCC-NCLSI [3,14,17,21,22,37,39], RIs for the 3 breeds (B, D, E) with small-sized samples could be calculated to validate existing RIs, since the reference samples were performed under the same local laboratory conditions of those used to transfer and validate the internal laboratory RIs.

## 3. Results

The percentage of observations falling outside the claimed RIs is reported in Table 1, which also includes the new RIs generated in this study. The species-specific RIs were generated following the latest recommendations [15,21,31,37,41]. Differences with RIs currently used in our laboratory (claimed RIs) and those previously published have been detected (Table 1 and Table 2).

Mean, standard deviation, median, maximum and minimum values are reported in Table 3.

**Figure 1:**
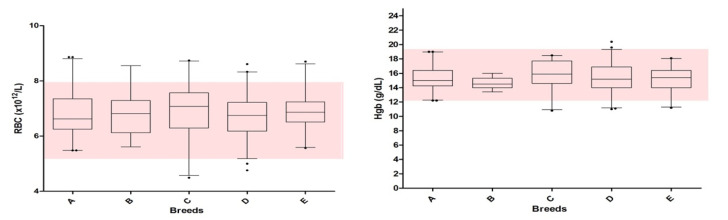

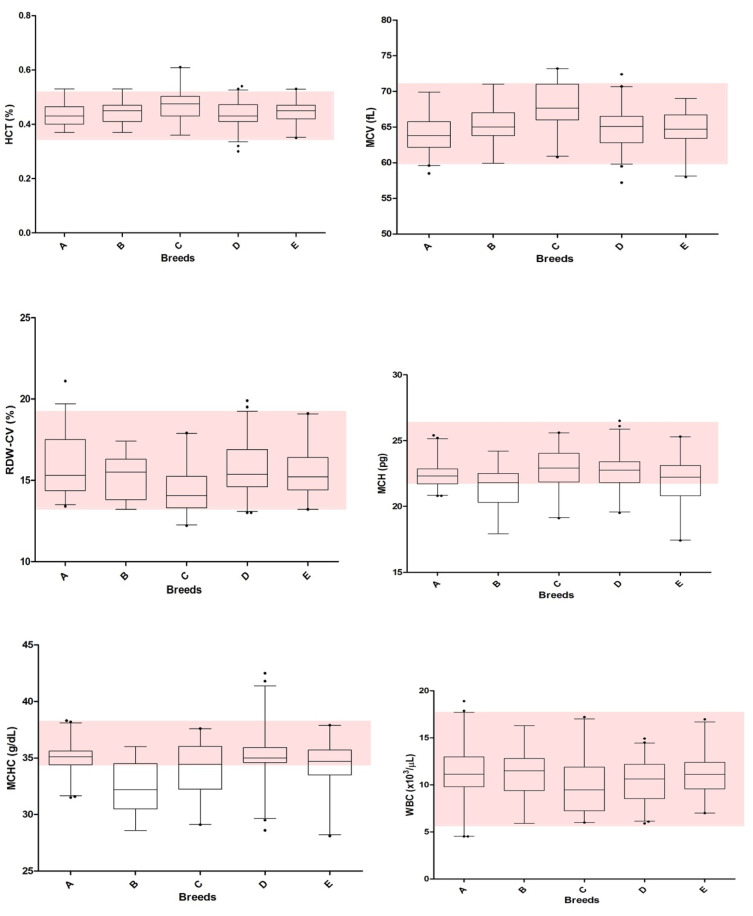

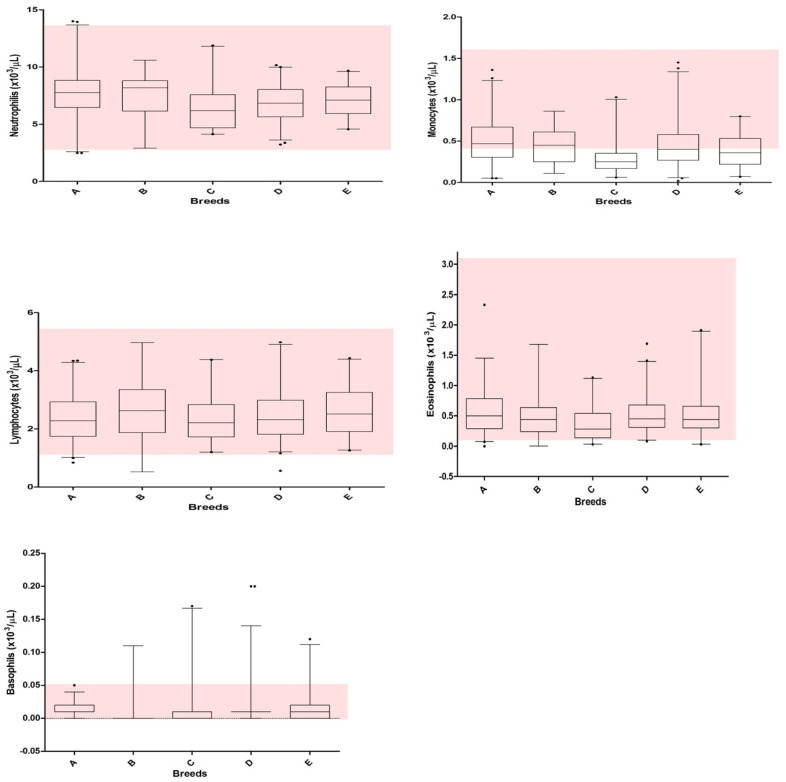
Hematological results recorded in the 5 breeds of dogs included in this study (156 Ariégeois–A, 52 Bleu de Gascogne–B, 64 Bracco italiano–C, 123 Segugio italiano–D, 50 Briquet Griffon Vandeen–E). The boxes represent the lower quartile, median, and upper quartile, and the whiskers represent the minimal and maximal values. The small points indicate outliers (i.e., values exceeding quartiles). The gray area indicates the reference intervals reported for hematological data (claimed RIs). (WBC = white blood cells; RBC = red blood cells; MCV = mean corpuscular volume; Hb = hemoglobin; HCT = hematocrit, MCHC = main corpuscular hemoglobin concentration; RDW-SD = red cell distribution width).

**Figure 2:**
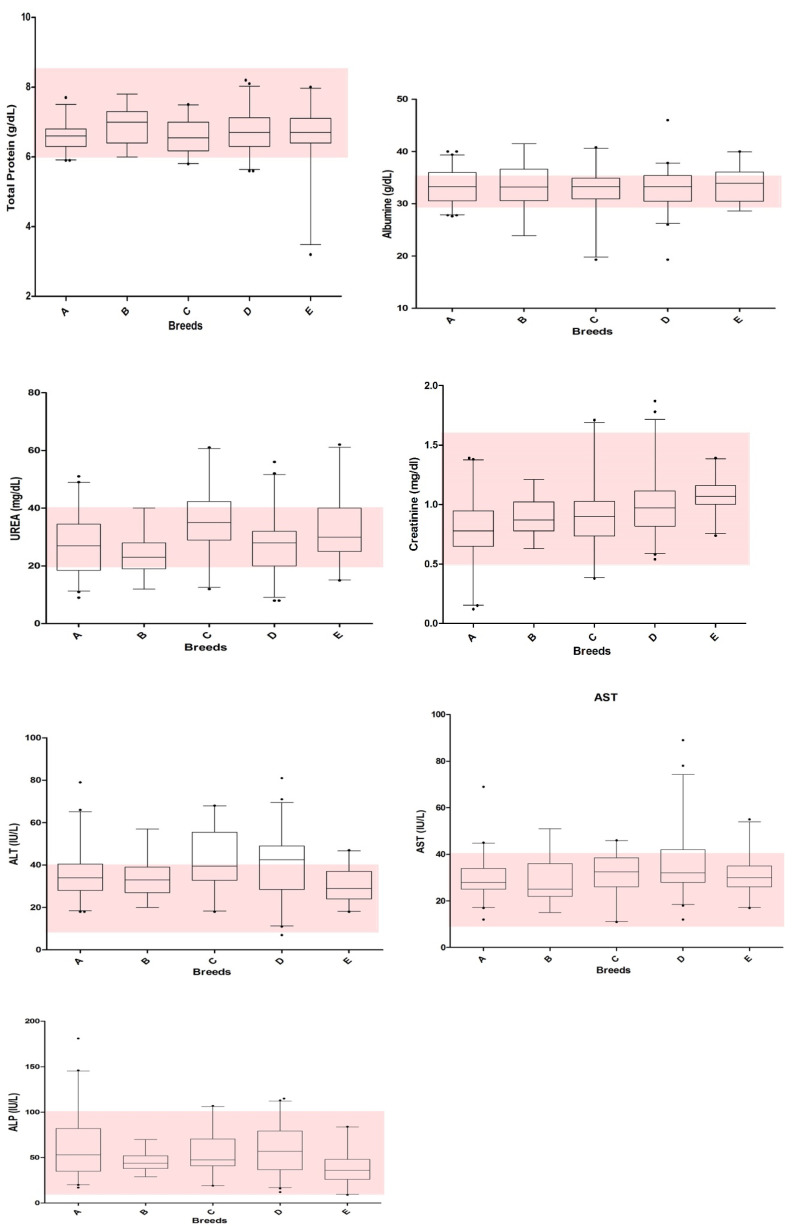
Biochemical results recorded in the 5 breeds of dogs included in this study (Ariégeois–A, Bleu de Gascogne–B, Bracco italiano–C, Segugio italiano–D, and Briquet Griffon Vandeen–E). The boxes represent the lower quartile, median, and upper quartile, and the whiskers represent the minimal and maximal values. The small points indicate outliers (i.e., values exceeding quartiles). The gray area indicates the reference intervals reported for biochemical data (claimed RIs) (Alb = albumin, TP = total protein, AST = aspartate aminotransferase, ALT = alanine aminotransferase, ALP = alkaline phosphatase).

The distribution of data recorded for each analyte is displayed in Figure 1 and Figure 2.

New RIs have been established for 12 parameters in at least 1 breed:

1. *Ariegois* dogs: New RIs were established for 7 analytes: MCH, MCHC, Monocytes, Albumin, Urea, Creatinine, and ALT.
2. *Bleu de Gascogne* dogs: New RIs were established for 9 analytes: MCH, MCHC, PLT, Monocytes, Eosinophils, Albumin, Urea, AST, and ALT.
3. *Bracco italiano* dogs: New RIs were established for 10 analytes: HCT, MCH, RDW, PLT, Monocytes, Eosinophils, Albumin, Urea, Creatinine, and ALT.
4. *Segugio italiano* dogs: New RIs were established for 7 analytes: MCH, Monocytes, Albumin, Urea, Creatinine, AST, and ALT.
5. *Briquet Griffon Vandeen* dogs: New RIs were established for 5 analytes: MCH, MCHC, PLT, Monocytes, and Albumin.

## 4. Discussion

The study demonstrates, for the first time, some hematobiochemical breed-specific peculiarities in 5 hunting–dog breeds. 

All hunting dog breeds had significant differences in concentrations for most hematobiochemical variables compared with “in house” and published RIs, which is in line with earlier studies (Table 1, Table 2 and Table 3) [14,15,31]. Obviously, our reference population of young adult dogs of medium-sized breeds may not be representative of the canine patient population, since age and breeds have a significant effect [31]. Interestingly, significant differences from the claimed RIs are shown in all or in most of these breeds for MCH, MCHC, PLT, absolute monocytes count, and Albumin, Urea, and Creatinine concentrations. It is unlikely that this was a result of physiological and aptitude factors, since age and sex distribution did not differ between the breeds. 

The hunting dog group has long been employed in assistance to hunters by pointing, retrieving, and flushing birds or hunting wild animals, reflecting strong behavioral pressures to locate and pursue quarry over great distances and variable terrain and climatic conditions. Hunting breeds have been adapted to their occupations by improved endurance, cardiac function, blood flow, and cognitive performance, demonstrating how strong behavioral selection alters physiology to create breeds with distinct capabilities [9,11]. The resulting breeds reflect the development of closed populations with strong phenotypic and genotypic homogeneity with well-defined physical and attitude attributes. 

For these reasons, breed-specific RIs should be recommended for these breeds with peculiar metabolic or physiological patterns raised from the adaptation to environmental conditions, to a specific aptitude (i.e., racing or hunting dogs) or to the effect of the generation of new breeds from pre-existing breeds [8].

The hunting breeds in this study included 3 French breeds (Ariegois, Bleu de Gascogne and Briquet Griffon Vandeen) and 2 Italian breeds (Bracco italiano and Segugio italiano), which are used for boar hunting and were selected for two main reasons. First, collaboration with local breeders and hunter’s associations allowed us to collect a sufficient number of samples to apply strict inclusion criteria for blood donors and enough residual data to establish RIs as recommended [21,22,37,38]; second, RIs are needed in these breeds, as no data is reported. 

The optimal method to develop reliable RIs is to sample (a priori) a large number of rigorously assessed healthy dogs of known age, sex, and breed, thus controlling for a number of pre-analytical variables [41]. For this purpose, the data obtained in our study using a large reference population of client-owned, clinically healthy blood-donor dogs represents a practical and effective indirect sampling method that ensures the homogeneity of the population [21,31,41]. We showed evidence of physiologic interbreed variation in a population representative of the true clinical picture. Extreme care has been taken to select those animals included as donors living in the same region (Umbria region, Central Italy) of the Veterinary Blood bank and the reference laboratory to standardize all possible variables so that an important environmental and geographic influence was avoided.

RIs generated in-house from a reference population of adult healthy individuals from the same geographical area, using the same instruments, reagents, and methods of validation as those on this study, have been used for comparison (claimed RIs) [21,22,30]. We preferred to follow this approach as, with a few exceptions, RIs from textbooks are usually very similar to those generated by any laboratory from the general hospital population, unless some patient peculiarity (e.g., breed, age range, aptitude) exists. Nevertheless, this approach may have induced some of the observed discrepancies because the reference population may differ from that of this study (e.g., our RIs have been generated from adult dogs, from 2 to 8 years old, while the general hospital population usually comprises also younger and older animals). For these reasons, for complete blood count, we used in-house hematological RIs transferred and validated from those published for the modern Laser–based Sysmex XT–2000iV analyzer [15], since it was the instrument used to obtain the CBC in all dogs included in the study. This analyzer has been validated for canine blood; its within-laboratory precision and repeatability has been assessed and is considered satisfactory, and it is more analytically sensitive for leukocyte count than manual counts, providing higher percentages than those reported in textbooks, given that flow cytometric methods analyze a much larger number of evenly distributed cells [31]. Recently, de novo hematology RIs for Sysmex XT were established for a general canine population in 2 studies on a large number of clinically healthy dogs [15,31].

In our study, hematological intervals for some parameters differed from recently published RIs for the Sysmex [12,14,15,31] (Table 3), and this is probably due to geographical and breed-specific differences as opposed to different adjustments or calibrations specific to each instrument, since these differences are evident in the majority of breeds evaluated [15]. This highlights the importance of RIs transference validation for pre-existing RIs by individual laboratories even when using identical instruments [18,31] and the need for setting breed-specific RIs. 

With regard to our CBC results, some breed peculiarities have been found: RIs for MCH and MCHC are both decreased in all 5 breeds compared to the claimed RIs even if not significantly for MCHC in Bracco italiano and Segugio italiano. These erythrocyte indices are calculated based on Hb concentration, RBC count, and HCT, so they amplify small deviations of these parameters. Indeed, even if not significantly, RIs for Hb tended to be lower and those for RBC count and Hct tended to be higher in these breeds (except for the HCT, which significantly increased in Bracco italiano) compared with the claimed RIs. Physiologically increased RBC count and HCT can result from dehydration, exercise, and work intensity, as well as stress mediated by splenic contraction [12,42]. However, in the current study, blood sampling was standardized, and animals stopped hunting activity at least 2 weeks before sampling. In these breeds, the tendency toward higher RBC mass (RBC counts and HCT) was offset by a tendency toward slightly lower Hb concentrations, MCH, and MCHC values compared with the general canine population, which may be a true physiologic characteristic of hunting dogs. Since all breeds had similar RIs for these analytes, it is more likely to reflect issues with the claimed and published RIs for the general canine population [15] and for mixed population blood donors [31] than pathology. As previously seen in Greyhounds and Lurchers [31], as well as in the agility dogs [18], adaptation to athleticism in breeds that excel at sports and hunting have experienced substantial genetic selective performance pressure on the blood circulation system to maximize the delivery of oxygen and metabolic substrates to exercising muscle [11,13,23,42,43]. 

In addition, 3 breeds (Ariegois, Segugio italiano and Briquet Griffon Vandeen) tended to have lower RIs for MCV, even if not significantly, than those claimed and published [14,15,18,31], which is indicative of their microcytic RBC compared to the general canine population. This could depend on the lower Hb content due to iron metabolism anomaly, nutritional deficiencies, intestinal disease, chronic inflammatory disease, intestinal parasitosis, or the increased physical activity typical of hunting breeds [44]. Iron concentrations and specific markers of iron deficiency were not measured in this study, since each dog donated blood only 1 time per year, and no signs of iron deficiency were recorded. MCV is highly related with RBC, Hct, and RDW and the size of the RBC is related to the number of RBC and Hct. Microcytosis not associated with anemia has been previously found in other canine breeds with RIs physiologically lower than the standard population [45] in dogs with abnormal RBC membrane structure or metabolism [46], or in humans with peculiar hemoglobin phenotypes [47]. Our results are in contrast with a previous study about microcytosis and MCV that did not find any statistical difference for Italian Hounds compared to the general population [45]. Conversely, Bracco italiano breed had slightly higher RIs for MCV compared to the general canine population [15]. These modifications of red cells parameters merit further investigations in hunting dogs. 

Interestingly, RIs for platelet count were higher in all breeds compared to the claimed RIs (significantly in 3 breeds) and to the published RIs for the Sismex XT–2000iV [12,14,15,18,31]. This instrument measures platelet concentrations with an optical method that appears to be strongly accurate [15]. Thrombocytosis at rest could be a secondary reactive process due to high serum cortisol levels related to stress, prolonged training activity, chronic inflammation and infectious disease, and iron deficiency other than being age-related [12,48]. Since all breeds studied tended to have high PLT count, it may be a physiologic feature of the reference population of adult hunting dog breeds. 

RIs for WBC count and for neutrophil, lymphocyte, monocyte, and eosinophil counts tended to be lower than the claimed RIs (even if significance was obtained only for monocyte and eosinophil counts) and the RIs published for the Sysmex–XT2000iV in the general canine population [15]. It has been demonstrated that WBC, lymphocyte, and neutrophil counts decrease significantly with age [15]. The reduced monocytes, lymphocytes, and eosinophils counts could also be related to stress response induced by long-term training activity and the environmental condition of housing in a shelter typical of these races [15,28,31]. It is important to state that most of the RIs for leukocytes (WBC in group A and WBC, neutrophils and lymphocytes in groups B, C, D, and E) were narrower than the claimed RIs, and the use of the newly established RIs might help to identify dogs that are erroneously considered healthy. On the other side, these parameters tended to be higher than those obtained using the Sysmex–XT2000iV in a mixed population of blood donors [31] and in other breeds [12,14,18]. 

Regarding the biochemical parameters, we used in-house RIs transferred and validated from those published in Kaneko [40]. All breeds showed wider RIs for Albumins with statistically significant lower low reference limits (LRL) and higher upper reference limits (URL) compared with those of the claimed RIs, as seen in trained agility dogs [49]. The reason for the increased albumin concentrations is unclear. On the other hand, RIs for Total proteins tended to be lower than the claimed RIs in all breeds as described in Greyhounds [13], even if not significantly. These results could suggest that globulin fractions were reduced in these races, and this should be deeply investigated by evaluating the serum protein electrophoresis. Serum protein concentrations could decrease during prolonged training in dogs because of increase plasma volume, exercise-induced immunodepression, catabolism of plasma proteins for energy, and protein loss via renal and gastrointestinal tracts [28].

Significantly different RIs were also identified for Urea and Creatinine concentrations in most of the hunting breeds evaluated compared with the claimed RIs. Particularly, Urea showed wider RIs with lower LRL in all breeds, lower URL in B breed, and higher URL in the A, C, D, and E breeds. The high concentration of Urea may depend on the inadequacy of RIs used in the internal laboratory or of those published [17,19,29]. On the other side, Creatinine had narrower ranges in B and E breeds, with decreased RIs in A, B, and E breeds and slightly increased RIs in C and D breeds (Italian hounds) compared with the claimed RIs and data published in other breeds [17,19,29]. Creatinine concentration provides the basis for the International Renal Interest Society (IRIS) [50]. The finding of high creatinine concentrations may have a significant effect on clinical decisions, especially when renal failure should be excluded. The current IRIS staging system classifies any dog with creatinine concentrations > 1.4 mg/dL as affected by renal azotemia. In the two breeds whose URL were >1.4 mg/dL but <1.7 mg/dL, a mild renal azotemia has been excluded, since clinical signs of chronic kidney disease were absent, and PU/CU were <0.2 in all animals included. If the unchanged glomerular filtration rate was assumed to have increased, creatinine concentrations might have been associated with a high-protein diet and with an enhanced creatinine production that has been found to be proportional to muscle mass. Particularly, the physiological variation of creatinine concentrations could be attributed to the variation of muscle mass and body stores of phosphocreatine, increasing (growth of muscles) and then decreasing with age (age-related sarcopenia) [13,29,42].

Regarding the enzymes ALT and AST, the RIs identified were higher (significantly in A, B, C and D breeds for ALT and in B and D breeds for AST) in all breeds than the claimed RIs and with those published in the agility dogs [28]. Even if not significantly, the RIs for ALP were increased for A, C, and D breeds and decreased for B and E breeds compared with the claimed RIs. 

Clinical signs potentially associated with muscular, hepatocellular, and biliary damage were never detected in the dogs, and drug administration was excluded. Therefore, in the absence of a reason for increased ALT, AST, and ALP, these findings could be considered nonbiologically relevant and physiologic features of these breeds. Furthermore, the RIs for these three enzymes tended to be similar or lower than those identified in other breeds (Bovaro del Bernese, Deerhounds and Dogue de Bourdeaux breeds) [17,19,29]. Physiological higher serum AST concentrations have been attributed to increasing age [10,20], increased muscle mass [13], and muscle catabolism during exercise [42]. Increased ALP activity may be related also to an active bone metabolism in adults. Interestingly, it has been demonstrated that the increased growth and improved function of skeletal muscle, which are controlled by specific genes (ABLIM3, CDH15), may have enhanced dogs’ physical fitness in hunting dog breeds. Moreover, the deletion of the ASICS3 gene in hunting breeds, as the result of an adaptive response, seems to prevent fatigue-enhanced muscle pain, therefore improving their muscle endurance ability [11]. 

The validation of internal and published RIs revealed that for a variable proportion of analytes, breed-specific RIs should be designed for the hunting breeds evaluated. However, in most cases, the difference between claimed and published and new RIs were minimal and not significant. The LRL and URL of the two RIs were very close to each other and likely due to the limitations of the internal RIs and those published, as mentioned previously. In this case, it would be more correct to use the new RIs, but the use of internal or published RIs would minimally affect clinical decisions. Conversely, if one or both limits of the new RIs were very different from the internal and published RIs and only a partial overlap exists, the new RI would be recommended.

In some cases, the distribution of data reported in Figure 1 (i.e., Hb, HCT, WBC, Neutrophils) and 2 (ALP) shows evidence of narrower ranges than the claimed RIs. This sometimes occurred for all the breeds (e.g., WBC), but it was seen for a specific parameter in a single breed (e.g., Hb in Bleu de gascogne). It is important to underline that the RIs established “in-house” or reported in the literature are sometimes wide, possibly reflecting a more varied population being used, so that most of the values recorded in the present study fell within pre-existing RIs. Furthermore, the use of a wider RI is not suggested, since this might represent a greater genetic variability and could allow including pathological samples as normal [40].

## 5. Conclusions

In conclusion, this study provides further evidence that significant interbreed differences exist concerning hematobiochemical parameters in dogs. Indeed, it demonstrates many clinicopathological peculiarities in 5 hunting dog breeds compared with claimed RIs previously determined for a general dog population, under similar conditions and with the same analyzer. We suggest new RIs for 12 analytes (HCT, MCH, MCHC, RDW, PLT, Monocytes, Eosinophils, Albumin, Urea, Creatinine, AST, and ALT) in at least one of the hunting dog breeds evaluated. For these analytes, breed-specific variation appears to be the most plausible explanation. These RIs could be useful in the future for the health and animal welfare of hunting dogs; they should be considered when interpreting laboratory data in both research and clinic decision-making. 

Moreover, this study provides breed-specific RIs for hunting dog blood donors, helping to avoid the misinterpretation of laboratory results in the selection of suitable blood donors. They can be directly used for analyses performed with the same analyzer or for other analytical methods after proper transfer and validation according to the ASVCP and IFCC–CLSI guidelines [21,39].

This first study regarding an analysis of 5 hunting breed-specific hematological and biochemical phenotypes highlights potential genetic components of hematobiochemical traits in this species and could suggest a genetic relationship between these breeds that should be supported by phylogenetic analyses of canine DNA [9,28].

## Figures and Tables

**Table 1:** Reported Reference Intervals (RIs) currently used in our laboratory (claimed RIs) and the new breed-specific RIs for each hunting dog breed (A: Ariegois; B: Bleu de gascogne; C: Bracco italiano; D: Segugio italiano; E: Briquet Griffon Vandeen). Statistical analysis was obtained after the removal of data that behave as outliers interpretable as aberrant observations. The first 20 items for each analyte were randomly selected and compared with the claimed laboratory RIs. The standard laboratory RIs were validated if 10% or less (0–2) of data fell outside the RI; on the other hand, they were rejected if >25% of data (5 or more) fell outside the RI. If 10–25% of data lay outside the laboratory RIs, the next 20 items on the list were selected and compared with the RIs as described above, using the threshold of 10% (% values in brackets) of data outside the RIs (*n* = 2) to validate or reject the RIs When the published RI was rejected, a new RI, reported in bold, was established following the ASVCP [21] and NCCLS [37] guidelines.

Parameters	A Ariegeois		B Bleu de Gascogne		C Bracco Italiano		D Segugio Italiano		E Briquet Griffon Vandeen		
	Outside RI (%)	Breed RI	Outside RI (%)	Breed RI	Outside RI (%)	Breed RI	Outside RI (%)	Breed RI	Outside RI (%)	Breed RI	Claimed RI for Dogs
**RBC** ×10^12^/L	5	5.48–8.8	5	5.61–8.29	10	4.57–8.71	5	5.18–8.32	5	5.58–8.61	**5.10–7.80**
**Hb** g/dL	5	12.2–18.95	0	13.4–16.0	0	10.9–18.4	5	11.1–19.3	15 (10)	11.2–18.0	**12.5–19.4**
**HCT** %	5	36.93–52.84	5	36.5–52.76	**15 (25)**	**35.91–60.58**	0	33.38–52.45	0	35.59–52.57	**34.0–51.5**
**MCV** fL	10	59.61–69.90	0	59.9–71.0	20 (10)	60.9–73.19	0	59.8–70.66	10	58.12–69.0	**60.0–72.0**
**MCH** pg	**30**	**20.8–25.1**	**50**	**17.9–24.2**	**25 (30)**	**19.1–25.5**	**15 (15)**	**19.5–25.8**	**50**	**17.4–25.2**	**22.1–26.5**
**MCHC** g/dL	**30**	**31.6–38.1**	**75**	**28.6–35.8**	**60**	29.1–37.5	5	29.6–41.3	**40**	**28.2–37.8**	**34.5–38.3**
**RDW-CV** %	5	13.5–19.7	0	13.2–17.1	**15 (20)**	**12.2–17.8**	0	13.0–19.2	0	13.2–19.0	**13.2–19.1**
**PLT** ×10^3^/L	10	200–537	**20 (20)**	**201–554**	**30**	**231–661**	0	193–659	**15 (15)**	**126–685**	**115–423**
**WBC** ×10^3^/uL	5	4.55–17.71	0	5.90–15.67	0	6.00–17.00	0	6.15–14.43	0	7.00–16.68	**5.60–17.80**
**Neutrophils** ×10^3^/uL	5	2.59–13.65	0	2.90–10.53	0	4.13–11.79	0	3.61–9.97	0	4.57–9.59	**3.20–13.40**
**Lymphocytes** ×10^3^/uL	5	1.02–4.27	5	0.52–4.84	0	1.20–4.37	5	1.21–4.90	0	1.27–4.39	**1.20–5.50**
**Monocytes** ×10^3^/uL	**45**	**0.04–1.23**	**45**	**0.11–0.84**	**85**	**0.06–1.00**	**60**	**0.05–1.34**	**70**	**0.07–0.79**	**0.50–1.80**
**Eosinophils** ×10^3^/uL	5	0.07–1.4	**25 (25)**	**0.00–1.68**	**15 (25)**	**0.03–1.11**	5	0.10–1.30	5	0.03–1.80	**0.15–2.90**
**Basophils** ×10^3^/uL	0	0.00–0.03	5	0.00–0.11	15 (10)	0.00–0.16	0	0.00–0.14	0	0.00–0.10	**0.00–0.05**
**ALB** g/dL	**45**	**2.78–3.91**	**50**	**2.39–4.15**	**35**	**1.98–4.05**	**45**	**2.62–3.77**	**45**	**2.86–3.99**	**2.90–3.50**
**TP** g/dL	10	5.90–7.50	0	6.00–7.80	10	5.80–7.49	0	5.63–8.02	0	6.00–7.98	**6.00–8.50**
**Urea** mg/dL	**45**	**11.3–48.85**	**30**	**12–39.1**	**20 (35)**	**12.52–60.7**	**25 (30)**	**9.12–51.62**	**35**	15.1–61.1	**20.0–40.0**
**Creatinine** mg/dL	**30**	**0.15–1.37**	5	0.63–1.21	**30**	**0.38–1.68**	**45**	**0.58–1.70**	10	0.75–1.38	**0.5–1.6.**
**AST** IU/L	10	17.1–44.7	**15 (20)**	**15–51**	10	11.15–45.92	**15 (25)**	**18.37–74.25**	0	17.1–53.9	**9.0–40.0**
**ALT** IU/L	**20 (15)**	**18.45–65,1**	**25 (15)**	**20–54**	**40**	**18,3–67,85**	**50**	**11.37–69.5**	5	18.1–46.7	**7.0–40.0**
**ALP** IU/L	5	20.3–145.4	0	29–69.1	0	19.2–106.3	10	17.1–112.2	0	9.5–83.8	**10.0–100.0**

RBC = red blood cells; Hb = hemoglobin; HCT = hematocrit; MCV = mean cell volume; MCH = mean cell hemoglobin; MCHC = mean cell hemoglobin concentration; RDW-CV = RBC distribution width–coefficient of variation; PLT = platelets; WBC = white blood cells; ALB = albumin; TP = total proteins; AST = aspartate aminotransferase; ALT = alanine aminotransferase; ALP = alkaline phosphatase.

**Table 2:** Reported RIs currently used in use in our laboratory (claimed RIs) and those previously published [12,14,15,18,31].

CBC	Claimed RIs for Dogs	RIs for General Dog Population (Bourges-Abella et al., 2011) [8]	RIs for Blood Donors’ Dogs (Serra et al., 2012) [22]	RIs for Greyhounds and Lurchers (Campora et al., 2011) [7]	RIs for Dogues de Bordeaux (Lavouie et al., 2014) [20]	RIs for Shetland Sheepdogs (Ruggerone et al., 2017) [11]
**RBC** ×10^12^/L	5.10–7.80	5.20–7.90	5.90–8.40	6.67–9.30	5.29–8.84	5.7–8.8
**Hb** g/dL	12.5–19.4	12.4–19.2	14.2–20.0	16.6–22.6	12.8–20.5	12.9–18.4
**HCT** %	34.0–51.5	35.0–52.0	43.0–60.0	49.0–65.0	34.8–55.7	37.0–57.0
**MCV** fL	60.0–72.0	60.0–71.0	65.0–79.0	65.4–77.2	61.7–73.2	60.0–77.0
**MCH** pg	22.1–26.5	21.9–26.3	22.0–25.9	22.9–26.4	22.6–26.1	––––
**MCHC** g/dL	34.5–38.3	34.4–38.1	30.8–35.5	32.0–36.0	34.6–38.1	31.0–36.0
**RDW–CV** %	13.2–19.1	13.2–19.1	12.9–18.3	–––	13.5–19.0	11.9–18.5
**PLT** ×10^3^/L	115–423	108–562	102–282	89–237	87–328	57.70–487.80
**WBC** ×10^3^/uL	5.60–17.80	5.60–20.40	5.6–14.50	3.60–8.40	6.74–17.31	6.0–19.5
**Neutrophils** ×10^3^/uL	3.20–13.40	2.90–13.60	3.10–9.40	3.60–8.40	4.39–12.11	3.0–11.5
**Lymphocytes** ×10^3^/uL	1.20–5.50	1.10–5.30	1.50–4.30	0.57–2.24	1.09–4.08	1.0–4.8
**Monocytes** ×10^3^/uL	0.50–1.80	0.40–1.60	0.10–0.70	0.06–0.41	0.27–1.18	0.1–1.5
**Eosinophils** ×10^3^/uL	0.15–2.90	0.10–3.10	0.20–1.40	0.00–0.43	0.25–3.26	0.1–1.2
**Basophils** ×10^3^/uL	0.00–0.05	0.00–0.05	–––	–––	–––	–––

CBC = complete blood count; RBC = red blood cells; Hb = hemoglobin; HCT = hematocrit; MCV = mean cell volume; MCH = mean cell hemoglobin; MCHC = mean cell hemoglobin concentration; RDW-CV = RBC distribution width–coefficient of variation; PLT = platelets; WBC = white blood cells.

**Table 3:** Mean, standard deviation, median, maximum, and minimum values for each hunting dog breed (A: Ariegois; B: Bleu de gascogne; C: Bracco italiano; D: Segugio italiano; E: Briquet Griffon Vandeen).

CBC	A Ariegeois Mean ± SD Median (Min–Max)	B Bleu de Gascogne Mean ± SD Median (Min–Max)	C Bracco Italiano Mean ± SD Median (Min–Max)	D Segugio Italiano Mean ± SD Median (Min–Max)	E Briquet Griffon Vandeen Mean ± SD Median (Min–Max)
**RBC**	6.86 ± 0.85	6.79 ± 0.67	6.95 ± 0.90	6.75 ± 0.76	6.87 ± 0.64
×10^12^/L	6.62 (5.48–8.86)	6.82 (5.61–8.55)	7.09 (4.49–8.74)	6.74 (4.76–8.61)	6.87 (5.57–8.70)
**HCT**	43.89 ± 4.39	44.40 ± 3.82	47.10 ± 5.58	43.66 ± 4.43	44.50 ± 4.08
g/dL	43.40 (36.70–52.90)	45.00 (36.50–52.60)	47.70 (35.90–60.70)	43.30 (30.40–53.90)	44.80 (35.40–52.60)
**Hb**	15.36 ± 1.74	14.68 ± 0.77	15.85 ± 1.89	15.38 ± 1.93	15.15 ± 1.72
%	15.00 (12.20–19.00)	14.50 (13.40–16.00)	15.90 (10.80–18.50)	15.20 (11.00–20.40)	15.40 (11.20–18.10)
**MCV**	63.95 ± 2.48	65.46 ± 2.80	67.80 ± 3.36	64.78 ± 2.84	64.73 ± 2.59
fL	63.80 (58.50–68.90)	65.00 (59.90–71.00)	67.60 (60.80–73.20)	65.10 (57.20–72.40)	64.70 (58.00–69.00)
**MCH**	22.37 ± 0.91	21.32 ± 1.79	22.84 ± 1.54	22.54 ± 2.52	21.81 ± 1.92
pg	22.3 (20.80–25.40)	21.80 (17.90–24.20)	22.90 (19.10–25.60)	22.75 (2.10–26.50)	22.20 (17.40–25.30)
**MCHC**	34.99 ± 1.20	32.54 ± 2.15	33.92 ± 2.38	35.22 ± 2.07	34.55 ± 2.19
g/dL	35.10 (31.50–38.30)	32.20 (28.60–36.00)	34.45 (29.10–37.60)	35.00 (28.60–42.50)	34.70 (28.10–37.90)
**RDW**	15.85 ± 1.87	15.32 ± 1.19	14.38 ± 1.45	15.73 ± 1.52	15.50 ± 1.46
%	15.30 (13.40–21.10)	15.50 (13.20–17.40)	14.05 (12.20–17.90)	15.35 (13.00–19.90)	15.20 (13.20–19.10)
**WBC**	11.31 ± 2.57	11.26 ± 2.17	9.72 ± 2.79	10.58 ± 2.25	10.90 ± 2.12
×10^3^/L	11.13 (4.52–18.90)	11.50 (5.90–16.30)	9.47 (6.00–17.20)	10.63 (5.90–14.92)	11.10 (7.00–16.96)
**Neutrophils**	7.68 ± 1.96	7.59 ± 1.80	6.52 ± 2.06	6.84 ± 1.60	6.99 ± 1.33
×10^3^/uL	7.73 (2.47–13.98)	8.16 (2.90–10.59)	6.17 (4.13–11.86)	6.84 (3.21–10.15)	7.10 (4.55–9.67)
**Eosinophils**	0.57 ± 0.38	0.51 ± 0.43	0.35 ± 0.27	0.52 ± 0.31	0.53 ± 0.40
×10^3^/uL	0.49 (0.00–2.32)	0.43 (0.00–1.68)	0.27 (0.03–1.12)	0.44 (0.08–1.69)	0.44 (0.03–1.90)
**Basophils**	0.01 ± 0.009	0.01 ± 0.03	0.02 ± 0.03	0.02 ± 0.02	0.01 ± 0.02
×10^3^/uL	0.01 (0.00–0.045)	0.00 (0.00–0.11)	0.007 (0.00–0.17)	0.01 (0.00–0.20)	0.01 (0.00–0.11)
**Lymphocytes**	2.37 ± 0.77	2.74 ± 1.04	2.39 ± 0.87	2.49 ± 0.89	2.61 ± 0.83
×10^3^/uL	2.27 (0.83–4.34)	2.63 (0.52–4.97)	2.21 (1.20–4.375)	2.32 (0.55–4.98)	2.50 (1.26–4.42)
**Monocytes**	0.51 ± 0.27	0.44 ± 0.22	0.27 ± 0.17	0.45 ± 0.28	0.38 ± 0.21
×10^3^/uL	0.47 (0.04–1.35)	0.44 (0.11–0.86)	0.24 (0.06–1.02)	0.40 (0.02–1.44)	0.36 (0.07–0.79)
**PLT**	312.45 ± 78.88	373.12 ± 89.82	386.44 ± 101.82	330.88 ± 95.97	336.41 ± 125.74
×10^3^/L	285 (187–552.00)	362 (201–557)	346 (230–664)	320 (191–698)	334 (126–691)
**Total Protein**	6.5 ± 0.4	6.9 ± 0.4	6.6 ± 0.4	6.7 ± 0.5	6.7 ± 0.4
g/dL	6.60 (4.30–7.70)	7.00 (6.00–7.80)	6.55 (5.80–7.50)	6.65 (5.60–8.20)	6.70 (6.00–8.00)
**Albumine**	3.33 ± 0.31	3.31 ± 0.41	3.29 ± 0.36	3.28 ± 0.35	3.36 ± 0.32
g/dL	3.32 (2.76–4.00)	3.32 (2.39–4.15)	3.33 (1.93–4.08)	3.33 (1.93–4.60)	3.39 (2.86–4.00)
**Urea**	27 ± 9	23 ± 6	36 ± 11	26 ± 9	32 ± 10
mg/dL	27 (9–51)	23 (12–40)	35 (12–61)	28 (8–56)	30 (15–62)
**Creatinine**	0.79 ± 0.27	0.91 ± 0.16	0.90 ± 0.27	0.99 ± 0.23	1.08 ± 0.13
mg/dL	0.78 (0.12–1.39)	0.87 (0.63–1.21)	0.90 (0.38–1.71)	0.97 (0.54–1.87)	1.07 (0.74–1.39)
**AST**	29 ± 7	27 ± 10	32 ± 8	35 ± 12	30.30 ± 7.56
IU/L	28 (12–69)	25 (15–51)	32 (11–46)	32 (12–89)	30 (17–55)
**ALT**	35 ± 10	32.93 ± 8.60	42.09 ± 13.57	39.79 ± 14.56	30.25 ± 7.62
IU/L	34 (18–79)	33 (20–57)	39 (18–68)	42 (7–81)	29 (18–47)
**ALP**	61 ± 33	45 ± 11	55 ± 21	59 ± 26	39 ± 18
IU/L	53 (17–181)	44 (29–70)	47 (19–107)	57 (12–115)	36 (9–84)

RBC = red blood cells; Hb = hemoglobin; HCT = hematocrit; MCV = mean cell volume; MCH = mean cell hemoglobin; MCHC = mean cell hemoglobin concentration; RDW-CV = RBC distribution width–coefficient of variation; PLT = platelets; WBC = white blood cells; ALB = albumin; TP = total proteins; AST = aspartate aminotransferase; ALT = alanine aminotransferase; ALP = alkaline phosphatase.
